# Adaptive changes in physiological and perceptual responses during 10-day heat acclimation training using a water-perfused suit

**DOI:** 10.1186/s40101-020-00217-x

**Published:** 2020-04-14

**Authors:** Yelin Ko, Seon-Hong Seol, Juho Kang, Joo-Young Lee

**Affiliations:** 1 Department of Textiles, Merchandising and Fashion Design, College of Human Ecology, Seoul National University, COMFORT Laboratory, Bld. # 222-Rm. # 306, 1 Gwanak-ro, Gwanak-gu, Seoul, 08826 Korea; 2 Department of Physical Education, Seoul National University, Seoul, Korea; 3 Research Institute of Human Ecology, Seoul National University, Seoul, Korea

**Keywords:** Heat acclimation, A water-perfused suit, Passive heat acclimation, Post-exercise passive heating, Whole-body sweat rate, Rectal temperature, Heart rate

## Abstract

**Background:**

While active heat acclimation strategies have been robustly explored, not many studies highlighted passive heat acclimation strategies. Particularly, little evidence demonstrated advantages of utilizing a water-perfused suit as a passive heating strategy. This study aimed to explore heat adaptive changes in physiological and perceptual responses during 10-day heat acclimation training using a water-perfused suit.

**Methods:**

Nineteen young males were divided into three experimental groups: exercise condition (*N* = 6, HA_EXE_, 1-h exercise at 6 km h^−1^ followed by 1-h rest in a sitting position), exercise and passive heating condition (*N* = 6, HA_EXE+SUIT_, 1-h exercise at 6 km h^−1^ followed 1-h passive heating in a sitting position), and passive heating condition (*N* = 7, HA_SUIT_, 2-h passive heating in a sitting position). All heating programs were conducted for 10 consecutive days in a climatic chamber maintained at 33 °C with 60% relative humidity. The passive heating was conducted using a newly developed water-perfused suit with 44 °C water.

**Results:**

Greater whole-body sweat rate and alleviated perceptual strain were found in HA_SUIT_ and HA_EXE+SUIT_ after 5 and/or 10 days (*P* < 0.05) but not in the exercise-only condition (HA_EXE_). Lower rectal temperature and heart rate were found in all conditions after the training (*P* < 0.05). Heat adaptive changes appeared earlier in HA_SUIT_ except for sweat responses.

**Conclusions:**

For heat acclimation in hot humid environments, passive and post-exercise heat acclimation training using the suit (water inflow temperature 44 °C) were more effective than the mild exercise (1-h walking at 6 km h^−1^). This form of passive heating (HA_SUIT_) may be an especially effective strategy for the elderly and the disabled who are not able to exercise in hot environments.

## Introduction

Water-perfused suits have been widely used in thermo-physiological studies to form an artificial microclimate on the skin for inducing cold stress [1], heat stress [2], or both [3]. Since circulating water temperature can be easily controlled inside such a suit to directly cool and/or heat the skin, this kind of clothing can be an effective whole-body hypothermia or hyperthermia induction method. Previous investigations on the thermo-physiological effects of water-perfused suits included focuses on age-related differences in cardiac functions [4], thermosensitivity of peripheral skin sites [5], vascular responses [6], and metabolic responses [7]. Furthermore, passive heating using the suit has also been investigated to determine whether it is effective for treating cancer [8] or symptomatic peripheral artery disease [9].

Despite its extensive use in manipulating skin and body core temperatures (*T*_core_), to the best of our knowledge, there is little research highlighting the potential benefits of utilizing a water-perfused suit as a heat acclimation (HA) strategy. Typically, exercising in heat (active HA strategy) is the most commonly accepted method to induce the following HA responses [10], all of which contribute to improved heat tolerance: (1) decrease in body core temperature; (2) reduction in heart rate; (3) lessened physiological strain index (PSI); (4) increase in sweat rate; (5) alleviated perceptual strain. Other studies advocated passive HA strategies, subjecting individuals to heat stress without any exercise mainly by immersion in hot water [11], entering a heat chamber [12], or sauna [13]. These studies suggested that active strategies are not necessary to induce HA as the main stimulus for heat adaptation is simply a repeated rise in body core temperature [14]. However, passive heating has been preferred less because it is not considered as effective as exercise to induce heat acclimation; thus passive heating has often been recommended post-exercise [14, 15].

Donning a water-perfused suit has proved a powerful method to elevate body core temperature which is the requirement for HA and may have the following advantages. First, during passive heating, heat flow through the suit can be continuously monitored. A vapor-impermeable layer, which can be an outer layer of the suit, makes it into a non-evaporative microclimate. Therefore, when heat is lost from the skin, it is considered to result only from dry heat transfer [16]. From calculated heat flow during passive HA training, individual heat storage can be estimated which may make comparisons between active and passive strategies easier. Second, donning a water-perfused suit may be a superior strategy in hot humid environments. According to Périard et al. [17], effects of humidity on HA are highly likely to exist because of the physiological and biophysical differences between the two kinds of heat. According to these authors, to attain a high rate of evaporative cooling in a hot humid environment, it is imperative to keep a higher skin temperature or a larger wetted skin area than in a dry environment. Both conditions can be readily met inside the enclosure of a skin-heating water-perfused suit, which might reinforce the rationale for choosing this newly proposed method to improve heat tolerance to humid summer climates, like those of South Korea or Japan. Last but not the least, development of a novel HA method with no exercise can expand the application of HA to the disabled or the old who are not in a condition to exercise in heat.

This study explored heat adaptive changes in physiological and perceptual responses during 10-day heat acclimation training using a water-perfused suit. Changes elicited by passive and post-exercise HA strategies utilizing a water-perfused suit were compared to those obtained by a mild exercise based, active strategy. The present study hypothesized that (1) donning a water-perfused suit as passive and post-exercise strategies would both induce heat adaptive changes during the assigned intervention, and (2) differential effects of HA induction between the passive and post-exercise strategies would exist.

## Methods

### Subjects

Nineteen young Korean males participated in the present study. The subjects were all non-athletes. Anyone having cardiovascular, respiratory, or heat-related illnesses or symptoms were excluded in the recruiting process. During the 10-day HA program, each subject was instructed to refrain from strenuous exercise as well as alcohol intake and not to eat any food before arriving at the laboratory. The aims, procedures, discomforts, and risks of the present study were explained to subjects before the first day of the experiments, and they signed to an informed consent form. The current study was approved by the Institute Review Board of Seoul National University (IRB No. 1905/003­008).

### Development of a water-perfused suit and the donning process

**Fig. 1:**
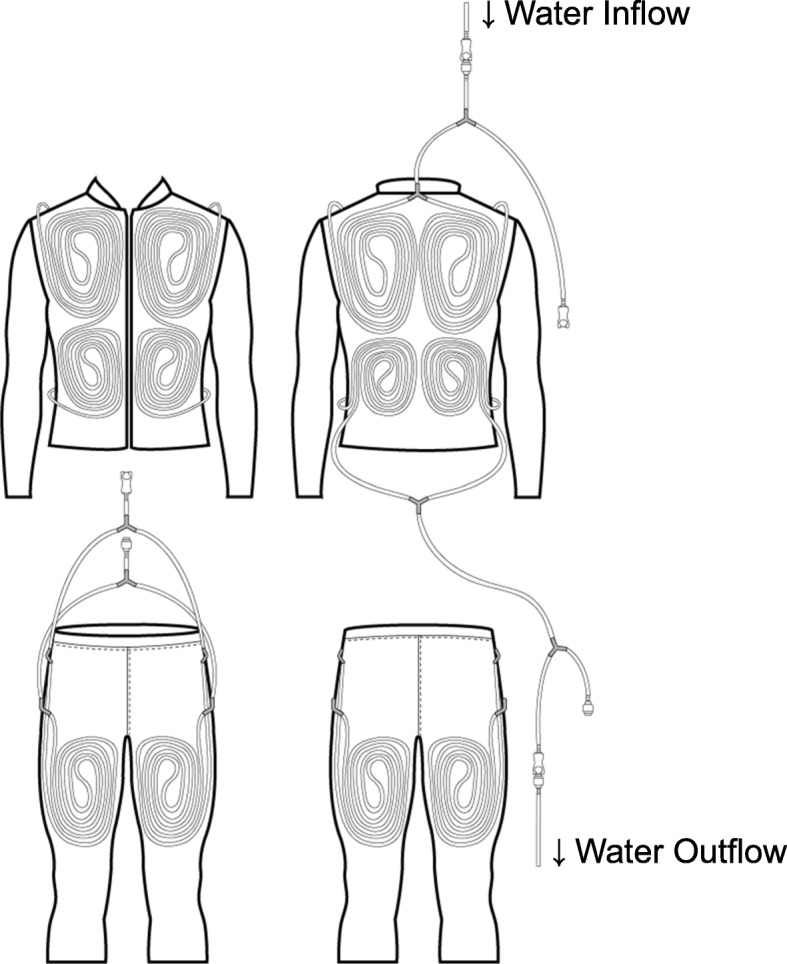
Structure of a developed water-perfused suit in the present study

A newly developed water-perfused suit was utilized in the experiments (Fig. 1). The suit consisted of a long-sleeve jacket and calf-length pants. The pants were cropped to make the donning process quicker. For easy washing, the inner layer (nylon-spandex mesh: nylon 85% and polyurethane 15%) and outer layer (polyester 92% and polyurethane 8%) were designed to be detachable. A total of 30.9 m of PVC tubing (inner diameter 4 mm and outer diameter 6 mm) was inserted into the inner layer in the chest, abdomen, upper and lower back, and front and back thigh. Peripheral body sites (hands, arms, legs, and feet) were not heated in order to optimize the water circulation rate. The suit was connected to a water bath circulator to heat the skin (RW-0525G, JEIO TECH, Korea, a resolution of 0.1 °C), with an average water flow rate of 37.31 ± 0.86 L h^−1^.

The donning process included the following steps: Firstly, after wearing the jacket and the cropped pants, subjects wore knee-length sports socks to cover their calves. Secondly, to enhance the efficiency of heat transfer from the circulating water to the skin, the outer layer of the jacket was pinned so that the suit tightly fit to each subject’s body. Two elastic bands (one around the chest and the other around the waist) were additionally worn to press the tubing against the skin. Thirdly, a vapor-impermeable insulating jacket and pants (polyester 100%, PVC coating) were donned over the suit to minimize evaporative and radiant heat loss from the body.

### Experimental conditions: 10-day heat acclimation intervention

The 10-day HA intervention consisted of three experimental conditions. Subjects were randomly assigned to one of the three experimental conditions and participated in the assigned 2-h program. Each subject was trained for their assigned experimental condition which they followed for their 10-day HA intervention. Subjects were evenly distributed in terms of age, height, body weight, body surface area, perceived heat tolerance, and maximal oxygen uptake (*V*O_2max_) into three groups (Table 1). The first group, HA_EXE_ (*N* = 6), was a mild exercise group. They completed a 1-h treadmill-walking exercise session at 6 km h^−1^ with 0% grade followed by a 1-h seated rest. The second group, HA_EXE+SUIT_ (*N* = 6), finished the same exercise session as HA_EXE_ did but further, post-exercise, donned the suit for another hour while sitting on a chair. The third group, HA_SUIT_ (*N* = 7), wore a water-perfused suit for 2 h without any exercise. The exercise intensity (6 km h^−1^ with 0% grade) for HA_EXE_ and HA_EXE+SUIT_ corresponded to 39 ± 4% HR_max_, which was estimated by the age of the subjects. Water inflow temperature (*T*_wi_) was 44.2 ± 0.1 °C for HA_EXE+SUIT_ and 44.3 ± 0.1 °C for HA_SUIT_ (*P* = 0.425).

**Table 1:** Characteristics of subjects in each experimental group

	HA_EXE_ (*N* = 6)	HA_EXE+SUIT_ (*N* = 6)	HA_SUIT_ (*N* = 7)	*P* value
Age (years)	22 ± 2	22 ± 2	24 ± 2	N.S.
Height (cm)	174 ± 2.2	174.8 ± 5.0	175.7 ± 6.0	N.S.
Body weight (kg)	69.5 ± 7.9	69.1 ± 10.2	71.7 ± 10.4	N.S.
Body surface area (m^2^)^a^	1.9 ± 0.1	1.9 ± 0.1	1.9 ± 0.1	N.S.
Perceived heat tolerance^b^	3 ± 2	3 ± 2	4 ± 1	N.S.
*V*O_2max_ (mL^·^min^·^kg^−1^)	47.5 ± 9.1	46.6 ± 6.8	47.5 ± 1.5	N.S.

All data were expressed as mean ± SD (standard deviation)

^a^Body surface area was estimated using the equation from Lee et al. [18]

^b^Perceived heat tolerance was attained with following 7-point scale: 1, I am very susceptible to heat; 2, I am susceptible to heat; 3, I am a little susceptible to heat; 4, I am neither susceptible nor tolerant to heat; 5, I am a little tolerant to heat; 6, I am tolerant to heat; 7, I am very tolerant to heat

### Experimental procedures

Each subject visited the laboratory in ten consecutive mornings to participate in the daily HA training program. Arriving at the laboratory at the scheduled time, subjects first took a rest drinking 300 mL of water. Identical, size appropriate undershorts, shorts, and socks were provided as experimental clothing before instrumentation. Subjects were instructed to bring their own running shoes every day. Subjects in HA_EXE_ and HA_EXE+SUIT_ waited in the preparation room wearing the set of experimental clothing before beginning their trial. By contrast, subjects in HA_SUIT_ took off their shorts and changed into a water-perfused suit approximately 10 min before their trial. For each trial, baseline rectal temperatures and heart rates were checked to make sure the values were within normal ranges before entering the climatic chamber (maintained at 33 °C with 60% relative humidity).

After 10 min of sitting, the 2-h HA program started according to each subject’s assigned experimental condition. For the first 1 h of the intervention, subjects in HA_EXE_ and HA_EXE+SUIT_ walked at an intermittent rate (2 repeats of 25-min walking and 5-min break). During the first break, they took a seated rest. After finishing the second walking session, during the second 5-min break, HA_EXE+SUIT_ changed into a water-perfused suit and started resting as soon as the change was complete, while HA_EXE_ maintained a standing posture before starting a seated rest for the remainder of the hour. No additional break to make up for the time it took for the change of clothes was not given to the subjects in HA_EXE+SUIT_ to equalize the duration exposed to the warm humid environment (2 h), between the groups. HA_EXE+SUIT_’s post-exercise passive heating session lasted for the second 1 h of the program (2 repeats of 25-min heating and 5-min break) and was initiated by connecting the circulating water bath to the suit to allow inflow of warm water. Under the post-exercise passive heating condition, a break was given by disconnecting the suit from the circulating water and by instructing the subjects to unzip the insulating jacket. Subjects in HA_SUIT_ experienced the passive heating protocol using a water-perfused suit for 2 h (4 repeats of 25-min heating and 5-min break). During the break, they took the same type of the break as HA_EXE+SUIT_ in the post-exercise heating session. All subjects drank a total of 600 mL of water ad libitum during the 2 h. It was not until the 2-h HA program was terminated that subjects were asked to leave the climatic chamber.

### Measurements and calculations

Rectal temperature (*T*_re_) was measured using a rectal probe inserted 16 cm beyond the anal sphincter and recorded every 5 s by a data logger (LT-8A, Gram Corporation, Japan). Heart rate was monitored every 1 s using a polar electrode with a chest belt (RC3 GPS, Polar Electro, Finland), and the data were sorted out at the interval of 5 s. Whole-body sweat rate was estimated using a change in total body mass which was measured in a semi-nude state on a calibrated scale before and after the experiment (ID2, Mettler-Toledo, Germany: resolution of 1 g). As all subjects were required to drink 600 mL of water in each trial, 600 g was subtracted from each subject’s post-experiment body mass. The body mass loss due to respiratory water loss was not considered significant. Perceptual responses were obtained every 10 min using the following scales: 9-point thermal sensation (4: very hot, 3: hot, 2: warm, 1: slightly warm, 0: neutral, − 1: slightly cool, − 2: cool, − 3: cold, and − 4: very cold) and 4-point thirst sensation (3: very thirsty, 2: thirsty, 1: a little thirsty, 0: not thirsty). Rating of perceived exertion (RPE) was obtained according to the Borg [19] scale, only for the HA_EXE_ and HA_EXE+SUIT_ because no exercise was required for HA_SUIT_. Physiological strain index (PSI) was calculated using the equation from Moran et al. [20] (Eq. 1). Peak PSI was calculated averaging the previous 5-min data at the time the maximum appeared. Heat flow (HF) through the water-perfused suit during the passive heating session was calculated using the following heat flow equation (Eq. 2). Heat flow was then converted to watts (1 kcal h^−1^ = 1.163 W).

1 $$ \mathrm{Physiological}\ \mathrm{strain}\ \mathrm{index}\ \left(\mathrm{PSI}\right)=5\left({T}_{\mathrm{re}\mathrm{t}}-{T}_{\mathrm{re}0}\right)\cdotp {\left(39.5-{T}_{\mathrm{re}0}\right)}^{-1}+5\left({\mathrm{HR}}_{\mathrm{t}}-{\mathrm{HR}}_0\right)\cdotp {\left(180-{\mathrm{HR}}_0\right)}^{-1} $$

where *T*_ret_ is rectal temperature at time *t* (°C), *T*_re0_ is rectal temperature at 0 min (°C), HR_t_ is heart rate at time *t* (bpm), and HR_0_ is heart rate at 0 min (bpm).

2 $$ \mathrm{Heat}\ \mathrm{flow}\ \left(\mathrm{HF}\right)\ \left(\mathrm{kcal}\;{\mathrm{h}}^{-1}\right)={h}_{\mathrm{w}}\cdotp {C}_{\mathrm{w}}\cdotp \left({T}_{\mathrm{w}\mathrm{i}}-{T}_{\mathrm{w}\mathrm{o}}\right) $$

where *h*_w_ is water flow rate of the suit (liters per hour), *C*_w_ is water specific heat = 1 kcal kg^−1^ °C^−1^, *T*_wi_ is water inflow temperature (°C), and *T*_wo_ is water outflow temperature (°C).

### Data analyses

All data were expressed as mean and standard deviation (mean ± SD). All statistical analyses were undertaken using SPSS statistics 25.0 with the significance level at *P* < 0.05. After testing for normality and sphericity, data from HA training on days 1, 5, and 10 were analyzed by using either a one-way repeated measures ANOVA or Friedman test for comparisons across the training days. As a post hoc test, pairwise comparisons with False Discovery Rate (FDR) correction were used when needed. When presenting results of rectal temperature increases during the three heat acclimation protocols, one-way ANOVA with the Tukey test as a post hoc was conducted to compare the values between the groups. The upper subscript a, b, and c in the sentences of the result section represented significantly identical experimental conditions determined by the Tukey test for post hoc. For graphical and analytical purposes, continuously measured rectal temperature and heart rate data during the training were averaged into 5-min blocks.

## Results

### Heat flow through the water-perfused suit and increment in rectal temperature during the intervention

During the first 1 h of the passive heating, heat flow through the water-perfused suit in HA_SUIT_ was 102 ± 8 W on average (Table 2). The 10-day average heat flow calculated during the second 1 h was 101 ± 9 W and 102 ± 7 W for HA_EXE+SUIT_ and HA_SUIT_, respectively (Table 2). One hour of mild exercise elevated *T*_re_ by 1.2 ± 0.2 °C^a^ for HA_EXE_ and 1.1 ± 0.2 °C^a^ for HA_EXE+SUIT_ respectively, while it was increased by 0.5 ± 0.2 °C^b^ by the passive heating in HA_SUIT_ (*P* < 0.001). During the second 1 h, *T*_re_ decreased by 0.5 ± 0.2 °C^c^ in HA_EXE_ while taking a seated-rest, it was further elevated by 0.5 ± 0.2 °C^b^ by post-exercise suit donning in HA_EXE+SUIT_, and in HA_SUIT_, it achieved 1.0 ± 0.1 °C^a^ (*P* < 0.001). As a result, net increase in rectal temperature was 0.7 ± 0.1 °C^b^, 1.6 ± 0.2 °C^a^, and 1.5 ± 0.2 °C^a^ for HA_EXE_, HA_EXE+SUIT_, and HA_SUIT_ respectively (*P* < 0.001). Average of area under the curve (AUC) of *T*_re_ for HA_EXE+SUIT_ (165.4 ± 15.7) was significantly greater than AUC of HA_SUIT_ (142.5 ± 20.6) (*P* = 0.048).

**Table 2:** Heat flow through the water-perfused suit on days 1, 5, and 10

	Day 1	Day 5	Day 10	10-day average	*P* value
Heat insertion by circulating water inside the water-perfused suit					
During the 1st 1 h of the suit donning (W)					
HA_SUIT_	101 ± 12	108 ± 9	100 ± 16	102 ± 8	N.S.
During the 2nd 1 h of the suit donning (W)					
HA_EXE+SUIT_	97 ± 16	106 ± 6	100 ± 16	101 ± 9	N.S.
HA_SUIT_	94 ± 16	107 ± 9	104 ± 12	102 ± 7	N.S.
*P* value	N.S.	N.S.	N.S.	N.S.	

All data were expressed as mean ± SD (standard deviation)

### Whole-body sweat rate

**Fig. 2:**
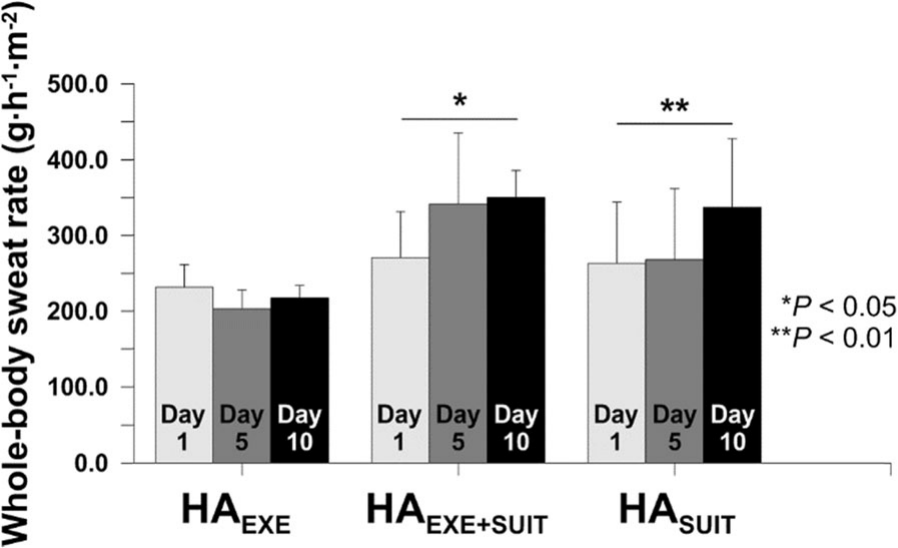
Whole-body sweat rate on days 1, 5, and 10 for HA_EXE_ (*N* = 6), HA_EXE+SUIT_ (*N* = 6), and HA_SUIT_ (*N* = 7)

Whereas HA_EXE_ did not show any differences in whole-body sweat rate across the HA training days, HA_EXE+SUIT_ and HA_SUIT_ displayed higher whole-body sweat rate on day 5 and/or day 10 (*P* = 0.013, *P* = 0.003, respectively) (Fig. 2). Compared to the first day of training, whole-body sweat rate in HA_EXE+SUIT_ on day 5 increased by 71.1 ± 51.5 g h^−1^ m^−2^ (*P* = 0.006) and on day 10 by 79.4 ± 44.5 g h^−1^ m^−2^ (*P* = 0.011) (Fig. 2). For HA_SUIT_, whole-body sweat rate increased on day 10: compared to day 1 it increased by 74.0 ± 51.7 g h^−1^ m^−2^ (*P* = 0.014) and to day 5 by 69.2 ± 45.3 g h^−1^ m^−2^ (*P* = 0.021).

### Perceptual responses

**Fig. 3:**
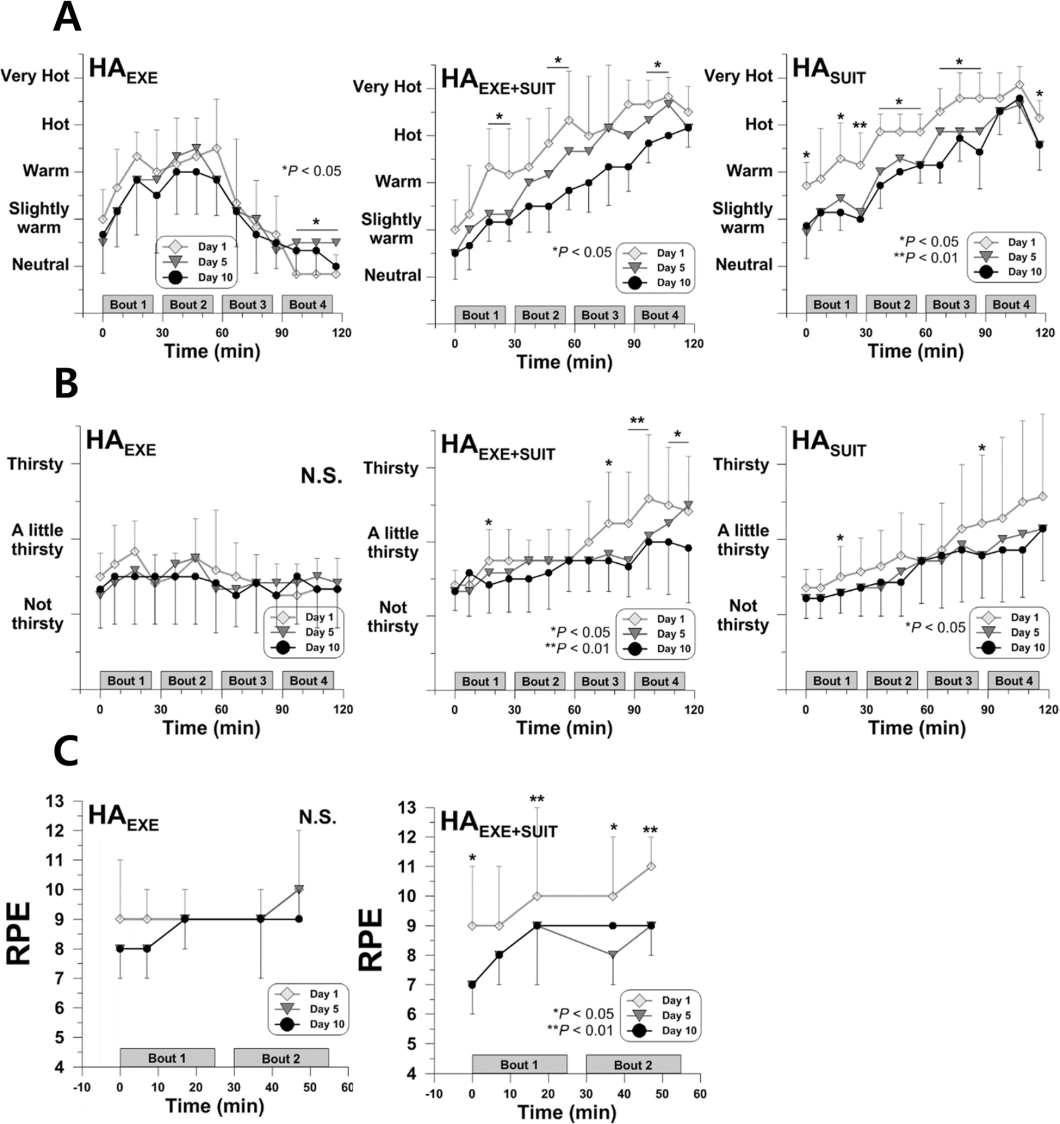
Thermal sensation (**a**), thirst sensation (**b**), and rating of perceived exertion (**c**) for HA_EXE_ (*N* = 6), HA_EXE+SUIT_ (*N* = 6), and HA_SUIT_ (*N* = 7)

The HA_EXE_ training adversely affected thermal sensation at the end of the seated rest, displaying lowest thermal sensation on the first day (*P* < 0.05) (Fig. 3a). In HA_EXE+SUIT_ and HA_SUIT_, on the contrary, subjects perceived themselves as being less hot after 10 days of HA training (*P* < 0.05) (Fig. 3a). Pairwise comparison with FDR correction showed that thermal sensation in HA_SUIT_ on day 10 was significantly lower than the first day (27th min, *P* = 0.035; 47th min, *P* = 0.042). Thirst sensation did not change in HA_EXE_ but decreased on day 5 and/or day 10 compared to day 1 in HA_EXE+SUIT_ and HA_SUIT_ (*P* < 0.05) (Fig. 3b). Rating of perceived exertion (RPE) showed no differences after 10 days of HA training in HA_EXE_. Subjects in HA_EXE+SUIT_, however, reported lower RPE on day 5 and/or day 10 during the exercise session (*P* < 0.05) (Fig. 3c). Pairwise comparisons with FDR correction showed that both on day 5 and day 10 RPE at 47th min for HA_EXE+SUIT_ significantly dropped from day 1 (*P* = 0.021 and *P* = 0.030, respectively).

### Rectal temperature

**Fig. 4:**
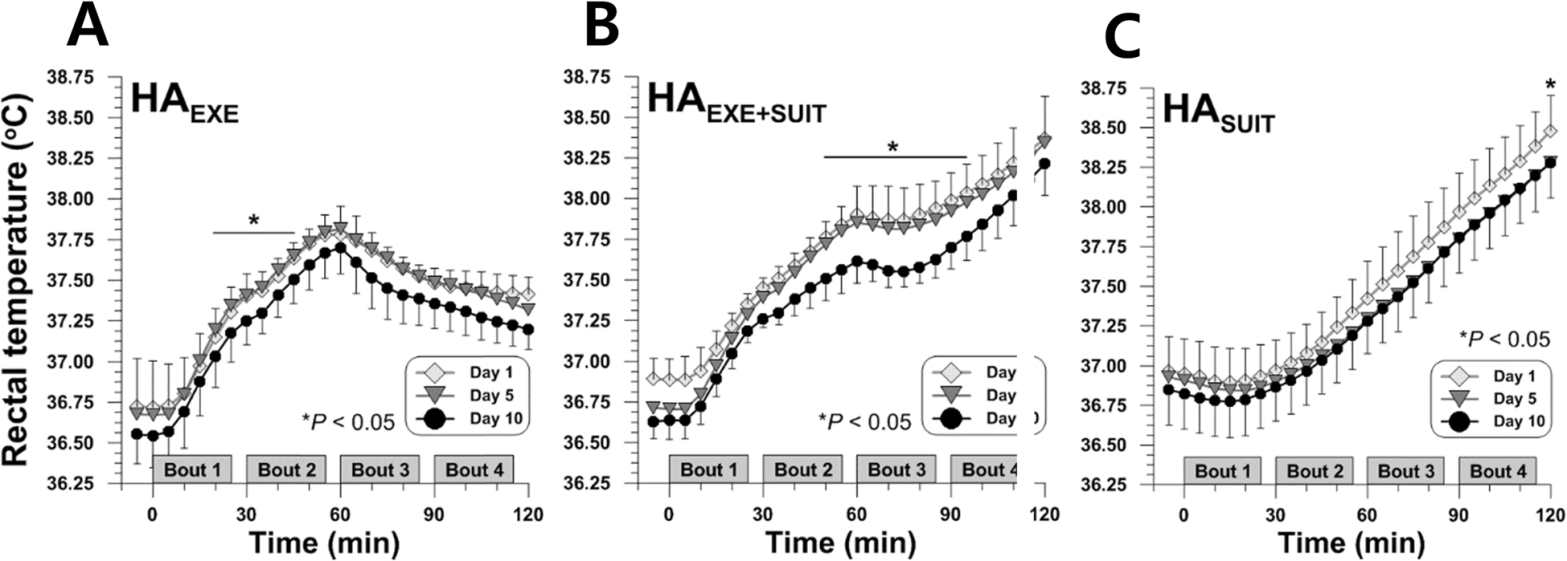
Time courses of rectal temperatures during heat acclimation training on days 1, 5, and 10 for HA_EXE_ (*N* = 6) (**a**), HA_EXE+SUIT_ (*N* = 6) (**b**), and HA_SUIT_ (*N* = 7) (**c**)

Lowest *T*_re_ appeared on day 5 or day 10 in the different stages of the training protocols for HA_EXE_, HA_EXE+SUIT_, and HA_SUIT_. The active strategy-based mild exercise group HA_EXE_ displayed lower rectal temperature on day 10 during the mid to last stage of exercise (*P* < 0.05) (Fig. 4a). Pairwise comparison with FDR correction revealed that in HA_EXE_, *T*_re_ of day 10 was lower than the values of day 1 (at 35th min, *P* = 0.038) and lower than those of day 5 (25th to 35th min, *P* < 0.05), respectively. For HA_EXE+SUIT_, significant differences across the days in *T*_re_ were present from the end of the exercise to the mid-stage of post-exercise suit donning (*P* < 0.05) (Fig. 4b). The HA_SUIT_ showed the lowest *T*_re_ at the end of the training (*P* < 0.05) (Fig. 4c).

### Heart rate

**Fig. 5:**
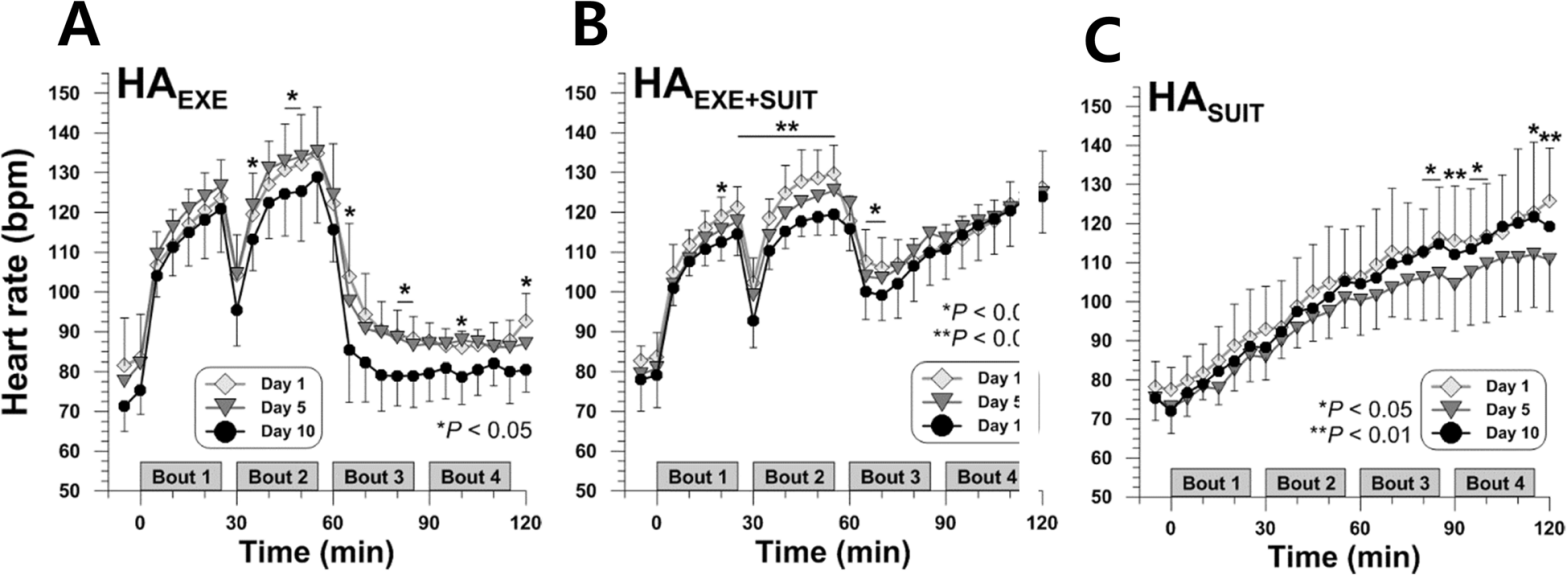
Time courses of heart rate during heat acclimation training on days 1, 5, and 10 for HA_EXE_ (*N* = 6) (**a**), HA_EXE+SUIT_ (*N* = 6) (**b**), and HA_SUIT_ (*N* = 7) (**c**)

Heart rate on day 10 for HA_EXE_ was significantly lower than day 1 and day 5 primarily during the seated rest period (*P* < 0.05) (Fig. 5a). According to the pairwise comparisons, heart rate for HA_EXE_ on day 10 was lower than on day 1 (at 50th, 65th, 80th, 100th, and 120th min, *P* < 0.05); it was also lower than heart rate on day 5 (at 100th min, *P* = 0.044). When passive skin-heating was added post-exercise (HA_EXE+SUIT_), heart rate on day 10 displayed lower values from the mid-stage of the exercise session to the earlier stage of passive HA session (*P* < 0.05) (Fig. 5b). The post hoc test showed that heart rate for HA_EXE+SUIT_ on day 10 significantly decreased from day 1 (20th to 55th and 70th min; *P* < 0.05). There were also significant differences in heart rate for HA_EXE+SUIT_ between day 1 and day 5 (40th and 45th min, *P* < 0.05). Unlike the other two HA strategies, HA_SUIT_ induced lower heart rate on day 5 at the later stage of skin-heating (*P* < 0.05) (Fig. 5c). Pairwise comparison with FDR correction indicated that on day 5 heart rate for HA_SUIT_ significantly dropped from day 1 (90th and 120th min, *P* < 0.05) and from day 10 (85th, 115th, and 120th min, *P* < 0.05).

### Physiological strain index (PSI)

**Fig. 6:**
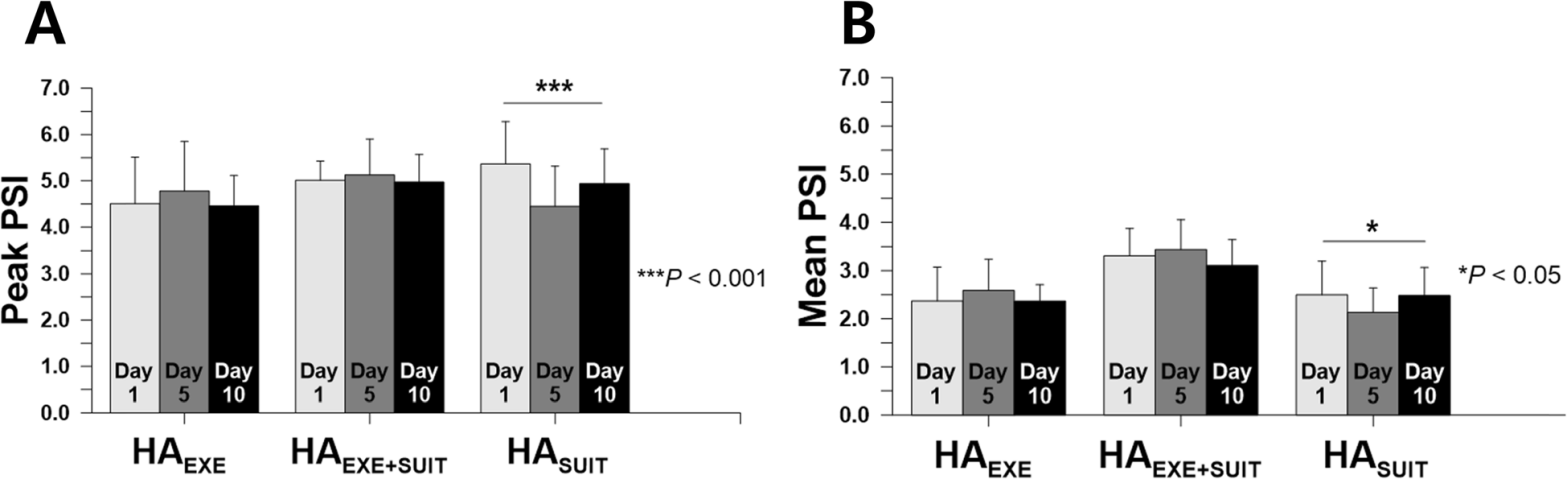
Peak Perceptual Strain Index (**a**) and mean Perceptual Strain Index (**b**) on days 1, 5, and 10 for HA_EXE_ (*N* = 6), HA_EXE+SUIT_ (*N* = 6), and HA_SUIT_ (*N* = 7)

Significant differences between the heat acclimation training days in peak and mean PSI were only found for HA_SUIT_ (Fig. 6a, b). Peak PSI for HA_SUIT_ on day 5 was the lowest (*P* < 0.001), showing significant differences from the values on day 1 (*P* = 0.001) and on day 10 (*P* = 0.018) (Fig. 6a). Compared to the other two days, mean PSI also displayed the lowest value on day 5 (*P* = 0.027) (Fig. 6b).

## Discussion

Human body can suffer from physiological and perceptual burdens in a hot environment in which the body temperature has to be effectively regulated, to prevent heat-related illness and impaired endurance performance [10, 14]. Being considered one of the most important intervention to reduce physiological strain in heat, various effective HA protocols have been suggested mostly based on exercising strategies. The present study aimed to propose the novel method to induce heat acclimation without exercise, which was wearing a skin-heating water-perfused suit to provide exogenous thermal loads to the body. The effectiveness of wearing the suit was proved by exploring physiological and perceptual changes during the 10-day HA trainings. There are two main findings of the current study. Firstly, both wearing the water-perfused suit after the 1-h exercise and throughout the 2-h protocol had more advantageous effects on inducing HA than the mild exercise HA strategy. Secondly, the HA responses that appeared in the passive and post-exercise HA groups differed in terms of the timing of the HA protocols and the training days.

### Beneficial effects of passive and post-exercise heat acclimation training using a water-perfused suit

When interpreting the present results, no significant differences in resting rectal temperature after training should be considered. Though the two HA strategies using a water-perfused suit induced “relatively” better responses than the mild exercise did, the extent of the obtained responses could be less than those reporting such differences. Whereas the mild exercise group (HA_EXE_) did not display any adaptive changes in whole-body sweat rate or perceptual responses, the other two HA strategies utilizing a water-perfused suit (HA_EXE+SUIT_ and HA_SUIT_) induced HA through those adaptive changes after 5 days and/or 10 days of training. Considering each subject drank exactly the same amount of water during the training, lessened thirst sensation despite enhanced sudomotor capacity obtained in HA_EXE+SUIT_ and HA_SUIT_ is particularly noteworthy. Sweating during exercise in heat was known to increase plasma osmolality accompanied by plasma volume loss, stimulating thirst sensation for unacclimated individuals [21]. Rise in plasma volume induced by heat acclimation, which was large enough to keep plasma osmolality low despite increased sweating may explain attenuated thirst sensation with higher sweat rates. Though plasma volume was not directly measured in the present study, it could be expected to have happened based on the high correlation of 0.93 between increased plasma volume and elevations in sweat rate [22].

The advantages of donning the skin-heating suit after exercise on HA responses are in accordance with previous studies: it was suggested that post-exercise hot water immersion [23] or sauna bathing [13, 24] were more effective HA strategies than exercise alone. It is also worthy to note that this strategy was more effective in alleviating perceptual thermal strain not only during the passive heating but also during exercise. Perceptual adaptations during exercise under heat stress are particularly important because those changes can relieve premature fatigue and exert positive influences on endurance performance [25–27]. In addition, heart rate for HA_EXE+SUIT_ was reduced after HA training primarily during exercise, while heart rate for HA_EXE_ decreased mainly during the recovery. Lower heart rates coupled with lower *T*_re_ found in HA_EXE+SUIT_ during exercise may be related with increased cardiac output, which can be possibly result from enhancement in plasma and stroke volume [28].

Passive strategies alone have been considered less effective for inducing HA than exercise-based strategies [10]. Even a review that highlighted the importance and practical values of passive HA protocols recommended these protocols be combined with exercise because exercise can lead to greater increases in body core temperature [14]. Daanen et al. [15] also pointed out that the positive adaptations induced by passive heat exposures, which are mainly related to blood volume, should be further compared and contrasted with actively induced HA responses. In contrast to the existing views, the findings of the present study suggest not only that donning a skin-heating suit for 2 h can be a sufficiently effective HA strategy but also that such a strategy may be more effective than mild exercise in heat for 1 h, at least in terms of activating sweat glands and alleviating perceptual strain as well as PSI. The effectiveness of wearing a water-perfused suit in inducing heat acclimation was, however, found to be better than only the mild exercise protocol walking at 6 km h^−1^ for 1 h. Further investigation is needed on how its effects will be compared to higher intensity exercise conducted for longer duration.

In order to match the heat stress of passive and active HA strategies, the heat flow through the water-perfused suit should be calculated. The approximate 100 W of heat flow during the 1 h of suit donning corresponds to the work rate that has been known to raise mean body temperature by 1.3 °C [29]. Given that *T*_re_ increased by 1.2 ± 0.2 °C during the first hour in HA_EXE_ while walking at 6 km h^−1^ with no incline, the difference between the heat storage rates in the passive versus active HA strategies of the current study does not seem too great. Still, whether the heat storage rates for each strategy should be matched is questionable considering passive strategies directly heat the skin first whose temperature rise would affect the temperature of body core whereas exercise-based strategies produce endogenous heat.

### Differential effects of passive and post-exercise heat acclimation training using a water-perfused suit

The two strategies heating the skin either after exercise or throughout the heat acclimation protocol induced HA responses at different times and on different acclimation days. In the exercise followed by passive HA, *T*_re_ and heart rate decreased mainly in the middle of the training while in the passive HA condition those values decreased at the end. Adaptive changes appearing later in the protocol for HA_SUIT_ are indeed not surprising considering that the increase in *T*_re_ for HA_SUIT_ during the first 1 h was only 0.5 ± 0.2 °C, but it was accelerated during the second 1 h reaching 1.0 ± 0.1 °C. When utilizing only a skin-heating suit, “sufficiently overloading thermal impulses” that exceed an adaptation threshold, which is known to be the requirement for HA [30] seems to be achieved by 2 h but not by 1 h. In addition, during the 2-h protocol AUC of *T*_re_ for HA_EXE+SUIT_ was significantly greater than that for HA_SUIT_, which may account for the earlier adaptation growth found during the protocol in HA_EXE+SUIT_.

Comparisons of the timing of HA induction in terms of reduction in *T*_re_ and heart rate during the protocol for HA_EXE+SUIT_ with those found in HA_EXE_ and HA_SUIT_ make it more reasonable to interpret that the changes are due to the combined effects of exercise and passive heating: it appeared from the end of exercise to the early stage of passive heating. During exercise, HA_EXE_ also elicited lower *T*_re_ and heart rate, as well known [31, 32], but it did not last (*T*_re_) or only intermittently appeared after exercise stopped (heart rate). The decreases of *T*_re_ and heart rate during the early phase of suit donning in HA_EXE+SUIT_ are less likely to be solely caused by the skin-heating, because in HA_SUIT_ such reduction in *T*_re_ and heart rate appeared at the end of the heat acclimation protocol. Therefore, it is more likely that as previously suggested [15, 23], the post-exercise passive heating further induced actively induced HA responses by maintaining greater thermal impulses after exercise using the suit. This synergic effect of exercise and suit donning seems to have facilitated improvement in heat loss mechanism leading to decreased heat storage [33] as well as in reduction of cardiovascular strain, whose mechanisms are known to be associated with (1) plasma volume expansion, (2) blood volume redistribution, (3) enhanced skin cooling, (4) increase in venous tone, and (5) less stimulated sympathetic nervous activity [17, 34].

Another point to bear in mind is that HA_SUIT_ showed adaptations earlier than HA_EXE+SUIT_ did except for the sweat responses. On day 5, HA_SUIT_ already showed reduction in *T*_re_ and heart rate contributing to reduction in PSI, in accordance with the findings of previous studies suggesting that less than 7 days of training are enough for body core temperature reduction [10, 35]; decrease in heart rate appears within the first 4–5 days [10, 17]. Lowest PSI on day 5 not on day 10 is likely to result from lower heart rate on day 5 than day 10, because *T*_re_ on day 5 and day 10 were not different. Seemingly diminishing effects of wearing the suit on inducing heat adaptive changes in heart rate may be attributable to the constant work-rate model of this HA strategy. Albeit practical, fixed endogenous, and exogenous thermal impulses have been pointed out to provide stimuli not strong enough for further adaptations, as HA gradually developed [30]. It will be worthy to be further investigated to examine whether reduction in heart rate obtained by wearing the suit adopting the controlled hyperthermia model, which is considered optimal in terms of addressing the aforementioned point, will not be attenuated.

The later expression of elevated whole-body sweat rate for HA_SUIT_ might be explicable by the warm humid microclimate formed inside the suit. When sweat adaptations occur at a peripheral level, it is seldom related to the number of activated eccrine glands but due to higher efficiency and larger sizes of sweat glands [36]. In a hot and humid environment, sweating adaptations are largely associated with hidromeiosis, which decreases the mean output of each sweat gland of wetted skin. Fox et al. [37] demonstrated that higher whole-body sweat rate induced in humid heat acclimation is linked with reduction in hidromeiosis. Candas et al. [38] also suggested that initial oversweating as well as delay in the occurrence of sweat reduction takes place in acclimated individuals. Therefore, it is probable that in the more poorly ventilated condition (HA_SUIT_) due to the longer duration of suit donning, the eccrine glands had to develop not only morphological and functional changes but also greater resistance to hidromeiosis, in order to show more profuse sweating. The different tendencies in *T*_re_ or heart rate (earlier in HA_SUIT_) and sweat response (later in HA_SUIT_) may be accounted for by the consensus that hidromeiosis and its adaptive changes are peripheral phenomenon and not related to central factors [38, 39]. Finally, 10 days is not a surprisingly long period for achieving an elevation in sweat rate as longer term HA interventions (at least 14 days were recommend by Tyler et al. [10]) have been shown to be appropriate for achieving sweat adaptations.

## Conclusions

We explored the effects of passive heating using a water-perfused suit on heat adaptive changes for 10 days. The novel finding of the present study is that the post-exercise and passive strategies that heated the skin using the suit (water inflow temperature 44.2–44.3 °C) was more effective than the mild exercise (1-h walking at 6 km h^−1^), in inducing adaptive changes in sweat and perceptual responses. In this regard, we can recommend to those who are not able to exercise in hot environments, utilize a water-perfused suit for at least 2 h daily for ~ 10 days to induce heat acclimation. The elderly or the disabled may be the population who can benefit from using this newly proposed heat acclimation protocol. However, it should be carefully applied to the old or further investigation for them is needed because the present findings were found for the younger subjects. Elite athletes who want intensified heat acclimation could also incorporate this novel method into their training programs. When choosing between passive and post-exercise strategies, optimal heat acclimation responses for the specific acclimation period should be considered.

## Data Availability

The datasets used and/or analyzed during the current study are available from the corresponding author on reasonable request.

## Abbreviations

HA: Heat acclimation
HA_EXE_: Heat acclimation with exercise
HA_EXE+SUIT_: Heat acclimation with exercise and post-exercise passive heating using a water-perfused suit
HA_SUIT_: Heat acclimation with passive heating using a water-perfused suit
HR: Heart rate
HF: Heat flow
PSI: Physiological strain index
RPE: Rating of perceived exertion
*T*_core_: Body core temperature
*T*_re_: Rectal temperature
*T*_wi_: Water inflow temperature
*T*_wo_: Water outflow temperature
*V*O_2max_: Maximal oxygen uptake
